# Decanoic acid, an MCT dietary component, alleviates cognitive impairment, cellular senescence, and promotes autophagy in accelerated aging and neurotoxic mouse models induced by chronic administration of D-galactose and D-galactose/AlCl_3_

**DOI:** 10.3389/fnagi.2025.1676926

**Published:** 2025-11-19

**Authors:** Shreshta Jain, Divya Vohora

**Affiliations:** Neurobehavioral Pharmacology Laboratory, Department of Pharmacology, School of Pharmaceutical Education and Research, New Delhi, India

**Keywords:** medium-chain triglycerides, aging, cognitive impairment, autophagy, senescence, amyloid toxicity

## Abstract

**Introduction:**

Cognitive decline advances with age, increasing the risk of dementia and Alzheimer’s disease among older adults. Medium-chain triglyceride (MCT) ketogenic diets have shown potential in slowing down age-related cognitive decline; however, the exact neuroprotective roles of MCT components, specifically decanoic acid and octanoic acid, remain unclear.

**Methods:**

Swiss Albino mice were subjected to D-galactose to trigger accelerated aging, or to a combination of D-galactose and aluminium chloride to mimic Alzheimer’s disease-like neurotoxicity. The animals received treated with decanoic acid, octanoic acid, or both. Cognitive function was assessed using the Morris water maze, while brain tissues were examined for oxidative stress markers, autophagy indicators, senescence activity, and amyloid-β levels.

**Results:**

Decanoic acid significantly improved learning and memory performance, enhanced antioxidant enzyme activity (superoxide dismutase, reduced glutathione, catalase), promoted autophagy by inhibiting mTOR, reduced cellular senescence (β-galactosidase-positive cells), and decreased amyloid-β toxicity. In contrast, octanoic acid showed no significant mechanistic effects, though it slightly improved cognitive behaviour.

**Discussion:**

This study demonstrates that decanoic acid, unlike octanoic acid, exhibits significant neuroprotective effects against accelerated aging and neurotoxicity, similar to Alzheimer’s disease. These findings highlight the differences in the neuroprotective mechanisms of decanoic and octanoic acids, implying that MCT-based diets should be re-evaluated as a preventive strategy for cognitive decline and neurodegenerative diseases.

## 1 Introduction

Cognitive aging is described as a gradual decline in brain functioning affecting memory, decision-making, reasoning, language and thinking abilities. Dementia is a severe case of cognitive deterioration that significantly affects a person’s daily quality of life. According to the WHO, dementia was the seventh leading cause of death in 2023 among the older population. The demographic data reports that by the year 2030, the population of people aged 60 years or above is expected to rise to 1.4 billion (World Population Prospect 2022, 2022). The prevalence of dementia, particularly Alzheimer’s disease, is projected to increase with the rapidly accelerating world’s elderly population (Alzheimer’s Association Report, 2024). Therefore, targeting biomarkers linked to aging could delay the onset of dementia related to Alzheimer’s disease and enhance health span and quality of life.

Biological aging occurs over a period of time and involves physical, physiological, metabolic and psychological alterations as a result of a continuous decline in the functioning of cells, tissues and organs (Kyriazis, 2020). The molecular and cellular factors that modulate the aging process include oxidative damage, degradation of autophagy, non-modulation of nutrient-sensing pathways, such as the mammalian target of rapamycin complex (mTOR) and AMP-activated protein kinase (AMPK) pathway, and cellular senescence (Campisi et al., 2019). Aging is also the most prominent risk factor associated with the onset of early Alzheimer’s disease. Literature evidence has demonstrated that the deposition of amyloid-*β* also impairs mitochondrial functioning, resulting in increased oxidative damage (Guo et al., 2022). Mitochondrial-derived reactive oxygen species (ROS) are known to induce autophagy by regulating the nutrient-sensing kinases, evidently through inhibition of mTOR and activation of AMPK (Gao, 2019). Autophagic elimination of damaged mitochondria (mitophagy) is also reported to be compromised (Guo et al., 2022). Impaired autophagy has been correlated to enhanced senescent burden as witnessed in various aging models of different species (Cassidy and Narita, 2022). Furthermore, reducing the burden of cellular senescence has been shown to improve cognitive aging and dementia related to Alzheimer’s disease (Chen et al., 2023; Liu et al., 2024; Stern, 2012).

Researchers have found evidence indicating that various nutritional interventions can reduce biomarkers of aging and lower the risk of age-related diseases such as Alzheimer’s disease (Bellantuono et al., 2020). Medium-chain triglycerides (MCT) are esterified medium-chain saturated fatty acids derived from natural sources such as coconut oil. These triglycerides break down into medium-chain fatty acids in the gastrointestinal tract and undergo *β*-oxidation in the liver to form ketones, including acetoacetate, beta-hydroxybutyrate (*β*HB) and acetone, as metabolites (Augustin et al., 2018). The MCT-supplemented ketogenic diet is extensively studied for its potential neuroprotective effects, which may counteract age-related neurological decline and disorders (Sun et al., 2023; Zhang et al., 2023). Recent research suggests it could be beneficial for elderly patients with Alzheimer’s disease and those experiencing cognitive decline due to aging (Dunn et al., 2023). A clinical trial with participants having mild cognitive impairment tested a ketogenic MCT drink, showing improved cognitive performance by offering an alternative brain energy source and bypassing glucose shortages (Fortier et al., 2019). The composition of MCT dietary supplements primarily includes decanoic acid (25–35%) and octanoic acid (65–75%) (Bach and Babayan, 1982). While the MCT diet has been investigated in clinical trials for its neuroprotective potential and cognitive benefits in Alzheimer’s patients, there have been no specific studies focusing on the roles of decanoic acid and octanoic acid in aging or age-related neurotoxicity. Preclinical studies indicate that decanoic acid may have neuroprotective effects in drug-resistant epilepsy by directly inhibiting AMPA receptors (Chang et al., 2015). It also shows promise in neurodegenerative diseases such as Alzheimer’s disease and Parkinson’s disease, by decreasing oxidative stress and serving as an alternative energy source for the brain fuel (Dunn et al., 2023; Sanguanphun et al., 2022). Nonetheless, the underlying biochemical pathways responsible for these phenomena remain elusive.

In this study, we aimed to expand current knowledge by assessing the neuroprotective effects of decanoic acid and octanoic acid on cognitive impairment caused by accelerated aging and age-related neurotoxicity. To our knowledge, this is the first research to investigate the individual neuroprotective roles of these medium-chain triglyceride (MCT) components against neurodegeneration linked to aging, using chronic D-galactose (D-gal) administration, which mimics natural aging symptoms and Alzheimer’s disease (AD)-like neurotoxicity induced by the combined chronic administration of D-gal and aluminium trichloride (AlCl_3_). The D-gal model is known to simulate accelerated aging through oxidative stress induction and mitochondrial dysfunction (Azman and Zakaria, 2019; Zhang et al., 2017; Cui et al., 2006), while the D-gal/AlCl₃ model reproduces Alzheimer’s disease features such as amyloid plaque deposition, cholinergic system disruption, and memory deficits (Luo et al., 2009; Peng et al., 2015). Using both models allowed us to determine if decanoic and octanoic acids consistently provide neuroprotective benefits in situations related to aging and Alzheimer’s disease.

Our findings offer new insights into how these fatty acids influence antioxidant activity, autophagy, cellular senescence, and amyloid toxicity.

## 2 Materials and methods

### 2.1 Animals

Swiss albino mice of both sexes, aged 8 weeks, were procured from the Central animal house facility, Jamia Hamdard, New Delhi, India, after gaining approval from the Jamia Hamdard Institutional Animal Ethics Committee (Registration no: 173/GO/Re/S/2000/CPCSEA; Registered on: 28th January, 2000) at the 67th meeting held on 23^rd^ May 2020 (protocol no. 1721). Animals were acclimatised for 7 days in the laboratory and were housed in polypropylene cages. All animals were maintained under controlled conditions of temperature and humidity (25 ± 2 °C, 55–65%) and a 12:12 h light–dark cycle, and they were provided with a standard pellet diet and had access to food and water ad libitum. All experimental procedures were carried out in accordance with the guidelines listed by the Committee for the Control and Supervision of Experiments on Animals (CCSEA), New Delhi, India. The presented study is reported following the ARRIVE (Animal Research: Reporting of *In Vivo* Experiments) guidelines. Following the Principles of the 3Rs- Replacement, Reduction, and Refinement, we minimised animal use by collecting behavioural data and brain tissue samples from the same animals employed in a prior study conducted in our lab (Jain and Vohora, 2025).

### 2.2 Drug treatment schedule

A total of 96 mice were randomly divided into eight groups, each for the D-galactose-induced accelerated aging model (Table 1), and D-galactose combined with AlCl_3_-induced age-related AD-like neurotoxic model (Table 2), whereby each group contained total number of 8 (*n*) mice (4 males, 4 females). The sample size of each group was calculated using G*Power software (version 3.1 for Mac). In the accelerated aging model, D-gal was given once daily via subcutaneous injection to Swiss albino mice for 8 weeks at a dose of 100 mg/kg (Kumar et al., 2009). To induce Alzheimer’s disease-related neurotoxicity, mice received daily oral doses of AlCl_3_ (20 mg/kg) and subcutaneous injections of D-gal (100 mg/kg) concurrently for 8 weeks (Xing et al., 2018). The doses of decanoic acid and octanoic acid were determined based on a clinical study involving patients with mild to moderate Alzheimer’s disease, which evaluated the effects of a ketogenic formula made from medium-chain triglycerides (MCTs) on cognitive functions (Ota et al., 2016). To estimate the corresponding doses for animals, the human doses were converted to animal equivalent dose using the Km ratio (Nair and Jacob, 2016). The calculated doses for mice were 1,100 mg/kg for decanoic acid and 1,400 mg/kg for octanoic acid. In the experiment, both compounds were administered together at their full individual doses, totalling 2,500 mg/kg. Each MCT component and its combinations were given once daily through oral gavage in a volume not exceeding 10 mL/kg of body weight, simultaneously with D-gal and D-gal/ AlCl_3_. They were administered as a suspension in a 0.5% aqueous methyl cellulose solution, which functions as a suspending agent to ensure consistent dosing and has no physiological or behavioural effects since it is not absorbed systemically.

**Table 1:** Drug dosage regimen for D-galactose induced accelerated aging in mice.

S. No. (*n* = 8)	Groups	Treatment (× 8 weeks)
I.	Control	0.9% saline solution
II.	Dg	D-gal s.c. (100 mg/kg)
III.	Dg + DA	D-gal s.c. (100 mg/kg) + DA p.o. (1,100 mg/kg)
IV.	Dg + OA	D-gal s.c. (100 mg/kg) + OA p.o. (1,400 mg/kg)
V.	Dg + DA + OA	D-gal s.c. (100 mg/kg) + DA p.o. (1,100 mg/kg) + OA p.o. (1,400 mg/kg)
VI.	DA	DA p.o. (1,100 mg/kg)
VII.	OA	OA p.o. (1,400 mg/kg)
VIII.	DA + OA	DA p.o. (1,100 mg/kg) + OA p.o. (1,400 mg/kg)

Dg/D-gal, D-galactose; DA, decanoic acid; OA, octanoic acid, p.o., per oral; s.c., subcutaneous.

**Table 2:** Drug dosage regimen for D-galactose with AlCl_3_ induced age-related Alzheimer’s disease-like model.

S. No. (*n* = 8)	Groups	Treatment (× 8 weeks)
I.	Control	0.9% saline solution
II.	Dg + Al	D-gal s.c. (100 mg/kg) + AlCl_3_ p.o. (20 mg/kg)
III.	Dg + Al + DA	D-gal s.c. (100 mg/kg) + AlCl_3_ p.o. (20 mg/kg) + DA p.o. (1,100 mg/kg)
IV.	Dg + Al + OA	D-gal s.c. (100 mg/kg) + AlCl_3_ p.o. (20 mg/kg) + OA p.o. (1,400 mg/kg)
V.	Dg + Al + DA + OA	D-gal s.c. (100 mg/kg) + AlCl_3_ p.o. (20 mg/kg) + DA p.o. (1,100 mg/kg) + OA p.o. (1,400 mg/kg)
VI.	DA	DA p.o. (1,100 mg/kg)
VII.	OA	OA p.o. (1,400 mg/kg)
VIII.	DA + OA	DA p.o. (1,100 mg/kg) + OA p.o. (1,400 mg/kg)

Dg/D-gal, D-galactose; Al, aluminium chloride (AlCl_3_); DA, decanoic acid; OA, octanoic acid; p.o., per oral; s.c., subcutaneous.

Throughout the experiment, no deaths occurred among any of the groups; all mice successfully finished the study, and their data were included in the analysis.

### 2.3 Behavioural analysis

#### 2.3.1 Morris water maze test

During the last week of the dosing schedule, all the animals from each group were allowed to perform a modified version of the Morris water maze task to test their memory and spatial learning (Zameer and Vohora, 2017; Vorhees and Williams, 2006). The test included a training period of 5 days followed by a probe trial test on the 6^th^ day. The Morris water maze consisted of a circular pool with dimensions-150 cm in diameter, 50 cm in height and was divided into four quadrants using four equally distanced sites, namely north, east, west, and south, along the edge of the pool. The pool was filled with water not exceeding the temperature of 25 ± 2 °C, and a platform made of transparent material with a width of 10 cm was placed in the pool, slightly submerged under the level of the water surface. The platform was used as the escape for the mice from the water maze.

For the memory testing phase of the test, the escape platform was placed randomly inside any of the four quadrants of the pool and was not removed throughout the duration of the test. The mice were trained for 5 days with 4 trials in each session, whereby the mice were placed in each quadrant as the starting point. The trials had a ceiling time of 60 s and an interval of 30 s between each trial. The time taken by the animals to locate the hidden platform, also known as escape latency, was recorded in seconds.

For the second phase of the task that evaluates spatial learning, a probe test was conducted after 24 h of the training phase. The platform was removed, and the mice were allowed to swim for 60 s, and the percentage of time spent (% dwell time) in the targeted quadrant where the platform was previously placed, was analysed.

### 2.4 Estimation of ketone body levels

At the end of 8 weeks, after completing the dosage treatment, urinary ketone levels were measured using Keto-Diastix reagent strips (Bayers). Urine samples were collected directly from each animal. The animals were gently restrained, and if they did not urinate spontaneously, light pressure was applied to the caudal abdomen to induce urination. A fresh urine drop was then placed on the reagent strip, and the colour change was assessed.

### 2.5 Sample collection

Post-completion of the investigation of the neurobehavioural parameters, the mice were euthanised using a CO_2_ inhalation chamber. The whole brain samples from every mouse were dissected and randomly divided for biochemical estimation and histopathological analysis, with each sample being stored separately. The whole brains were stored at −20 °C for biochemical estimation, and for histopathological analysis, they were fixed in 10% formalin solution.

### 2.6 Estimation of oxidative stress markers

Whole brains were homogenised in phosphate buffer solution (pH 7.4) and centrifuged at 13,000 g for 10 min at 4 °C. The supernatants were collected and used for the estimation of oxidative stress parameters. Protein estimation was performed using the assay method described by Lowry et al. (1951).

#### 2.6.1 Superoxide dismutase

The activity of antioxidant superoxide dismutase (SOD) was chemically assayed using the method provided by Kono (1978). The assay consisted of preparing a reaction mixture containing 0.1 mM ethylenediaminetetraacetic acid (EDTA), 50 mM sodium carbonate, 1 mM hydroxylamine hydrochloride, 24 μM of nitro-blue tetrazolium (NBT) and 0.03% v/v Triton X-100. In a test tube, 2.93 mL of this reaction assay system was taken to which 0.07 mL of brain homogenate sample supernatant was added, and the change in optical density (OD) per minute was determined at a wavelength of 560 nm at a time interval of 60 s for up to 3 min. The principle of the assay is based on the inhibition of photoreduction of NBT in the presence of SOD; hence the % of inhibition of NBT by SOD was calculated, followed by the computation of SOD units (50% inhibition = 1 SOD unit) per mg protein (Zhang et al., 2016).


$$\begin{array}{l} \%\text{Inhibition of}\;\mathrm{NBT}\;\;\text{reduction}\;\mathrm{by}\;\mathrm{SOD}=\\ \frac{\begin{array}{l} \text{Change in}\;\mathrm{OD}/\min\;\text{of Control}-\\ \text{Change in}\;\mathrm{OD}/\min\;\text{of sample}\times 100 \end{array}}{\text{Change in}\;\mathrm{OD}\;\mathrm{per}\;\min\;\text{of Control}} \end{array}$$

#### 2.6.2 Reduced glutathione

The determination of reduced glutathione (GSH) was performed using Ellman’s reagent (Jollow et al., 1974; Sachdeva and Chopra, 2015). The assay method included precipitation of 1 mL of sample brain homogenate supernatant with 1 mL of 4% sulphosalicylic acid. The mixture was kept at 4 °C for 1 h, followed by centrifugation for 15 min at 1200 g. The reaction concoction consisted of 0.1 mL of supernatant from the previous mixture, 2.7 mL of 0.1 M phosphate buffer (pH 7.4), and 0.2 mL of 0.1 mM of Ellman’s reagent (5,5′-dithiobis-(−2-nitrobenzoic acid); DTNB). The absorbance of the yellow colour obtained was measured at the wavelength of 412 nm. The GSH content was quantified using the following formula (Ellman, 1959):


SHconcentration(sample)=(A/e)×D

$$=({\mathrm{Abs}}_{41\mathrm{2nm}}/13600)\times (\text{Total volume}/\text{Sample volume})$$

where, A = Absorbance at 412 nm (Abs_412nm_).

e = Extinction coefficient (13,600 M^−1^ cm^−1^ for DTNB).

D = Dilution factor (Total volume/Sample volume).

#### 2.6.3 Catalase activity

The catalase activity was determined in terms of *k* min^−1^, whereby *k* is the rate constant of the first-order reaction of the decomposition of hydrogen peroxide (H_2_O_2_) catalysed by the presence of the catalase enzyme (Cohen et al., 1970). In a 96-well plate, 4 μL of sample brain homogenate supernatant was incubated with 93 μL H_2_O_2_ for 3 min at 37 °C. The incubation reaction was stopped by adding 19 μL of 6 M sulphuric acid (H_2_SO_4_). To this, 130 μL of 1.9 mM of potassium permanganate (KMnO_4_) was added, and the absorbance was measured immediately at 490 nm using a microplate reader for 3 min at time intervals of 60 s. A blank was taken, consisting of phosphate buffer solution, H_2_SO_4_ and KMnO_4_. The catalase activity was concluded by calculating the rate constant *k* using the following formula (Del Maestro and McDonald, 1987):


$$k=(S_{0}/S_{3})\times 2.3t$$

where, k = the first-order reaction rate constant.

t = the time interval over which the reaction is measured (3 min).

S_0_ = absorbance of blank.

S_3_ = absorbance of samples subtracted from blank absorbance.

2.3 = the first-order kinetic conversion factor.

### 2.7 Estimation of amyloid toxicity through quantitative and qualitative assessments

The amyloid-*β* protein was quantitatively determined through ELISA in D-gal combined with AlCl_3_-induced age-related AD-like model as per the manufacturer’s protocol (Krishgen Biosystems, India). Furthermore, to qualitatively identify the amyloid aggregates, 10 μm thick slices of the cerebral cortex were sliced on a microtome and treated with Highman’s Congo Red stain. The amyloid deposits are identified by the red-stained cells (Yakupova et al., 2019).

### 2.8 Estimation of biochemical markers through total RNA isolation and RT-PCR

Total RNA was extracted from brain tissues using Trizol-C reagent (SRL, India) and analysed using a ND-1000 Spectrophotometer (NanoDrop), followed by reverse-transcription into cDNA using Verso cDNA Synthesis Kit (Thermo Fisher Scientific Inc., US). Quantitative RT-PCR was performed using the iTaq Universal SYBR Green Supermix (Bio-Rad Laboratories, United States) on a CFX connect Real-Time PCR system (Bio-Rad Laboratories, United States), as per the protocol provided by the manufacturer. The primers were designed using NCBI primer blast^1^ (Table 3). The amplification was performed with iTaq Universal SYBR Green Supermix with 0.4 μL of each forward and reverse primers for the target of interest and I μL of cDNA as template, using the following conditions: 95 °C for 20–30 s (polymerase activation), 35 to 40 cycles of 95 °C for 15 s (denaturation), and 60 °C for 30 s (annealing). After each cycle run, a melting curve analysis was performed, using the following conditions: 65 °C to 95 °C with 5 °C increment for 2–5 s per step. Raw Ct values were determined to analyse the relative fold change of target genes.

**Table 3:** List of mRNA primers.

Gene	Forward primer	Reverse primer
*mTOR*	5′- CCGCTACTGTGTCTTGGCAT-3′	5′- CAGCTCGCGGATCTCAAAGA-3′
*PRKAB*	5′- CGACCCCAGCGTCTTCAG-3′	5′- CCTTGCCTCCTTCAGACCAG-3′
*APP*	5′- TGCAGCAGAACGGATATGAGAAT-3′	5′- TCAAAAGCCGAGGGTGAGTAAA-3′
*Bace1*	5′- GGATTATGGTGGCCTGAGCA-3′	5′- CACGAGAGGAGACGACGATG-3’
*GAPDH*	5′- AGAAGGTGGTGAAGCAGGCA-3’	5′- CGAAGGTGGAAGAGTGGGAG-3’

### 2.9 Estimation of nutrient-sensing markers using ELISA

The mammalian target of rapamycin (mTOR) is known as the negative regulator of autophagy, while phosphorylated adenosine-monophosphate-activated protein kinase (p-AMPK) is termed the positive regulator. Both were estimated in brain homogenates using ELISA kits, respectively, as per the protocol provided by the manufacturer (Krishgen Biosystems, India).

### 2.10 Estimation of senescence *β*-galactosidase (SA-*β* gal) assay

Senescent cells were detected using a Senescence *β*-Galactosidase staining kit based on the upregulation of senescence-related *β*-galactosidase activity during aging. The senescent *β*-galactosidase catalyses the formation of a dark blue stain from the substrate X-Gal. The staining of 10 μm thick slices of the cerebral cortex was performed as per the instructions provided by the manufacturer of the assay kit (Abbkine Inc., United States). A positive result was characterised by blue colouration in the cytoplasm observed via optical microscopy. For each sample, four distinct, non-overlapping microscopic fields (about 100 cells) were analysed to determine the percentage of cells staining positive for senescence-associated *β*-galactosidase (SA-*β*-gal) across all experimental groups.

### 2.11 Statistical analysis

The data from the Morris water maze test was analysed by repeated measures two-way ANOVA followed by Tukey’s multiple comparison test. Other data was analysed using one-way ANOVA followed by Tukey’s multiple comparisons test. All the values are expressed as Mean ± SEM. The value of *p* < 0.05 is considered statistically significant. Analysis was performed using the software GraphPad Prism, version 9.5.0 for Mac. No significant differences were observed between male and female mice; hence, the data were aggregated and presented as combined results to enhance statistical validity.

## 3 Results

### 3.1 Effects of decanoic acid, octanoic acid and their combination on memory and spatial learning

**Figure 1:**
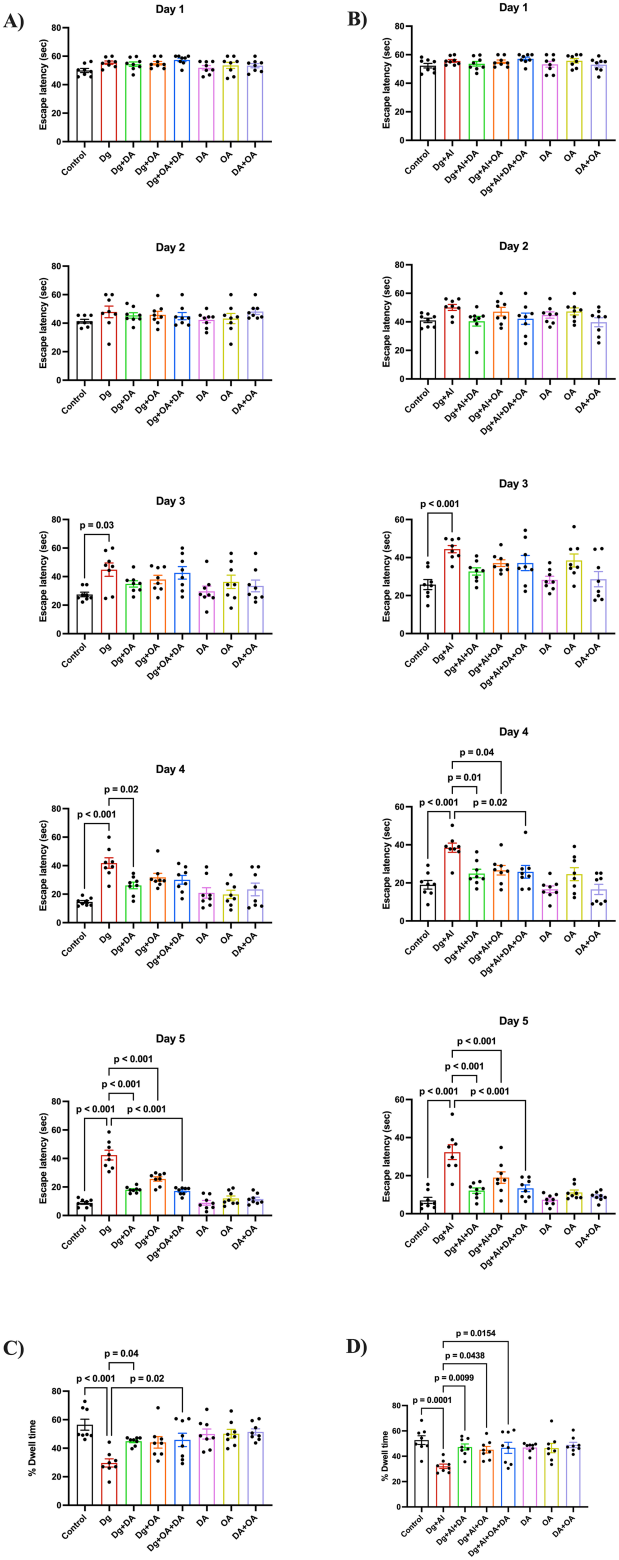
Memory and spatial learning based on the Morris water maze task: The figure shows **(A,B)** escape latencies to find the hidden platform during the training phase from Day 1 to Day 5, and **(C,D)** % of dwell time spent in the target quadrant during the probe trial, in the D-gal induced aging model and in the D-gal/AlCl_3_ induced AD-like neurotoxic model, respectively. Data is represented as Mean ± SEM (*n* = 8; 4 males, 4 females) and analysed by one-way ANOVA followed by Tukey’s multiple comparison test. Actual *p*-values for all comparisons are displayed. DA, decanoic acid; OA, octanoic acid; Dg, D-galactose, Al, aluminium chloride (AlCl_3_).

During the training phase of the Morris water maze task, it was observed that D-gal induced aged mice exhibited a significant increase their escape latencies indicating impaired learning behaviour on Day 3 (*p* = 0.03 versus Control), Day 4 (*p* < 0.001 versus Control) and Day 5 (*p* < 0.001 versus Control) (Figure 1A), which was significantly reduced in mice administered with decanoic acid on Day 4 (*p* = 0.02 versus Dg) and Day 5 (*p* < 0.01 versus Dg), octanoic acid (Day 5, *p* < 0.001 versus Dg) and their combination (Day 5, *p* < 0.001 versus Dg). In the probe trial, aged mice spent significantly less time in the target quadrant, implying a remarkable loss in spatial memory (*p* < 0.001 versus Control), which was significantly prolonged on administration with decanoic acid (*p* = 0.04 versus Dg) and its combination with octanoic acid (*p* = 0.02 versus Dg) (Figure 1C).

Similarly, AD-like neurotoxic mice (Dg + Al) exhibited significantly prolonged escape latencies on Day 3 (*p* < 0.001 versus Control), Day 4 (*p* < 0.001 versus Control) and Day 5 (*p* < 0.001 versus Control) and significantly diminished % dwell time (*p* < 0.001 versus Control). Administration with decanoic acid (*p* = 0.01 versus Dg + Al; *p* < 0.001 versus Dg + Al), octanoic acid (*p* = 0.04 versus Dg + Al; *p* < 0.001 versus Dg + Al) and their combination (*p* = 0.02 versus Dg + Al; *p* < 0.001 versus Dg + Al) on Day 4 and Day 5, significantly shortened the escape latencies (Figure 1B), while loss in % dwell time (*p* = 0.0001 versus Control) was also significantly reversed by decanoic acid (*p* = 0.0099 versus Dg + Al), octanoic acid (*p* = 0.0438 versus Dg + Al) and their combination (*p* = 0.0154 versus Dg + Al) (Figure 1D). Analysis of swim speed revealed no significant differences among the groups during both the training and test phases (data not shown).

### 3.2 Effects of decanoic acid, octanoic acid and their combination on urinary ketone levels

**Figure 2:**
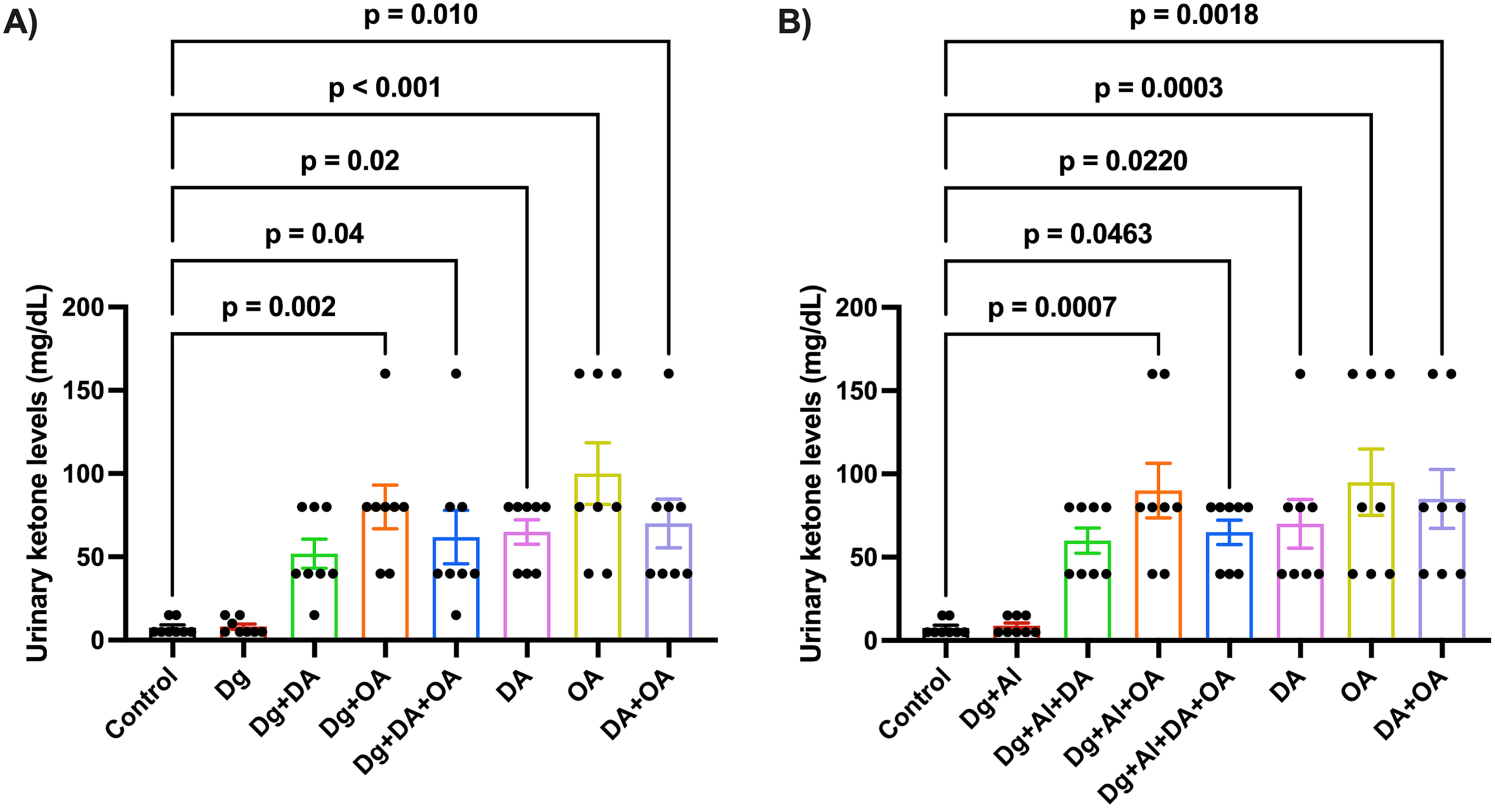
Urinary ketone body levels: The figure depicts the ketone body levels estimated by urinary analysis of mice in **(A)** D-gal induced accelerated aging model and **(B)** D-gal/AlCl_3_ induced AD-like neurotoxic model, respectively. Data is represented as Mean ± SEM (*n* = 8; 4 males, 4 females) and analysed by one-way ANOVA followed by Tukey’s multiple comparison test. Actual *p*-values for all comparisons are displayed. DA, decanoic acid; OA, octanoic acid; Dg, D-galactose, Al, aluminium chloride (AlCl_3_).

The analysis of urinary ketone levels is expressed in Figures 2A,B. Administration of decanoic acid, octanoic acid and their combination significantly increased the levels of ketone bodies in healthy mice (*p* = 0.02/*p* = 0.0220 versus Control; *p* < 0.001/*p* = 0.0003 versus Control; *p* = 0.01/*p* = 0.0018 versus Control, respectively). In accelerated aged mice, administration of octanoic acid (*p* = 0.002 versus Control) and its combination with decanoic acid (*p* = 0.04 versus Control) showed a significant rise in urinary ketone levels, whereas decanoic acid alone did not. Consequently, mice with induced Alzheimer’s disease-like neurotoxicity displayed a significant elevation in ketone levels when supplied with octanoic acid (*p* = 0.0007 versus Control) and its combination with decanoic acid (*p* = 0.0463 versus Control).

### 3.3 Effects of decanoic acid, octanoic acid and their combination on the parameters of oxidative stress

**Figure 3:**
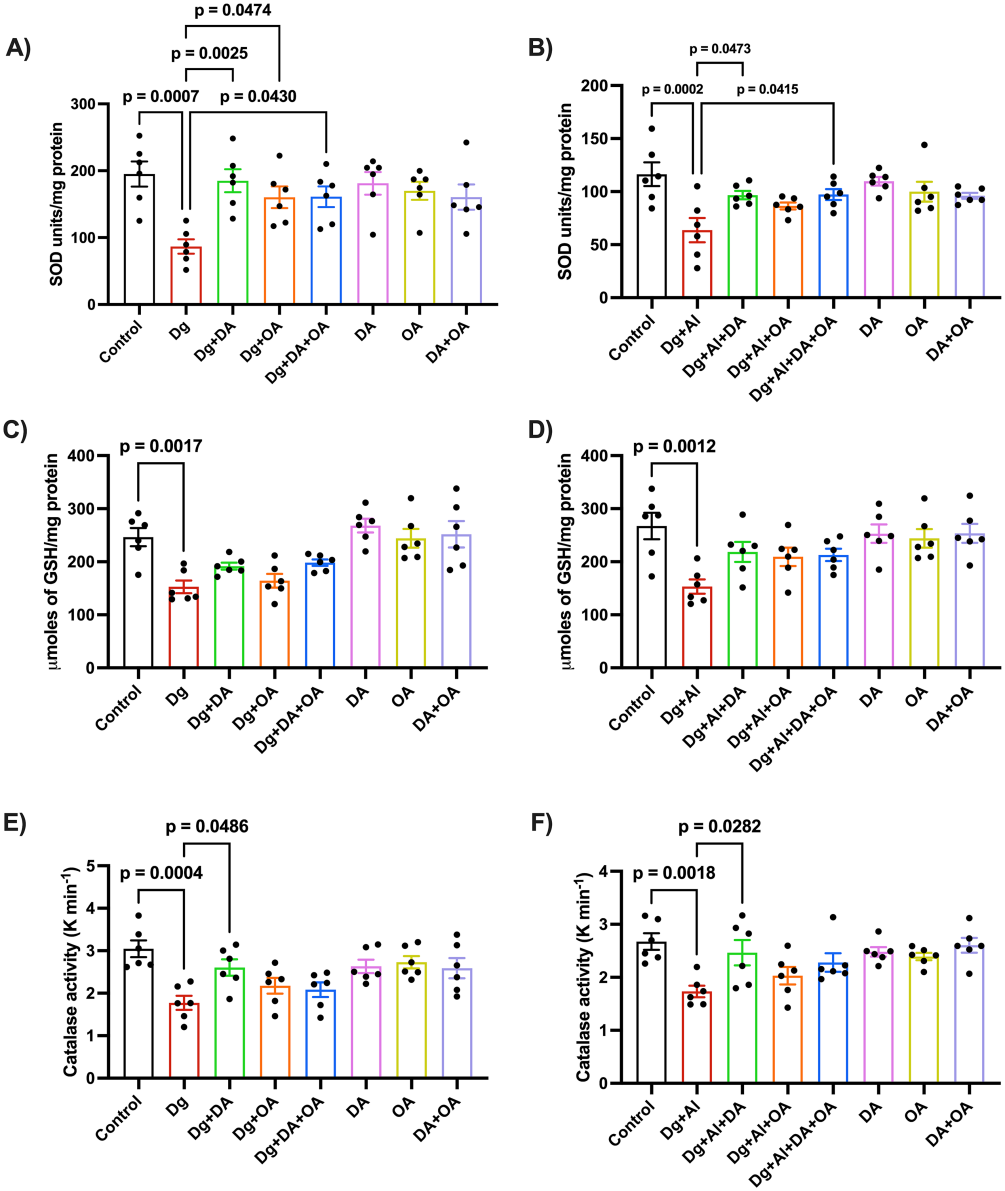
Evaluation of oxidative stress: The figure represents effects of MCT components on **(A,B)** superoxide dismutase (SOD), **(C,D)** reduced glutathione (GSH) and **(E,F)** catalase (CAT) in the D-gal induced aging model and in the D-gal/AlCl_3_ induced AD-like neurotoxic model, respectively. Data is represented as Mean ± SEM (*n* = 6; 3 males, 3 females) and analysed by one-way ANOVA followed by Tukey’s multiple comparison test. Actual *p*-values for all comparisons are displayed. DA, decanoic acid; OA, octanoic acid; Dg, D-galactose, Al, aluminium chloride (AlCl_3_).

The Figure 3 showcases the results after analysing the levels of anti-oxidants including SOD, reduced GSH and catalase (CAT), in the D-gal induced aged mice as well as in the age-related AD-like model induced by D-gal/AlCl_3_. A significant decrease in the activity of the anti-oxidant enzymes- superoxide dismutase (SOD), catalase (CAT) and glutathione (GSH), is observed in D-gal model (*p* = 0.0007 versus Control; *p* = 0.0017 versus Control and *p* = 0.0486 versus Control, respectively) and in D-gal/AlCl_3_ induced neurotoxic model (*p* = 0.0002 versus Control; *p* = 0.0012 versus Control and *p* = 0.0018 versus Control, respectively). Decanoic acid (*p* = 0.0025 versus Dg; *p* = 0.0473 versus Dg + Al, respectively), octanoic acid (*p* = 0.0474 versus Dg), and their combination (*p* = 0.0430 versus Dg; *p* = 0.0415 versus Dg + Al, respectively), exhibited reversal of the decline in the SOD enzymatic action; however, only decanoic acid increased the loss in catalase activity as observed in both the accelerated aging and age-related neurotoxic models (*p* < 0.0486 versus Dg; *p* = 0.0282 versus Dg + Al, respectively).

### 3.4 Effects of decanoic acid, octanoic acid and their combination on the quantitative and qualitative analysis of amyloid toxicity in D-gal/AlCl3 induced AD-like neurotoxic model

**Figure 4:**
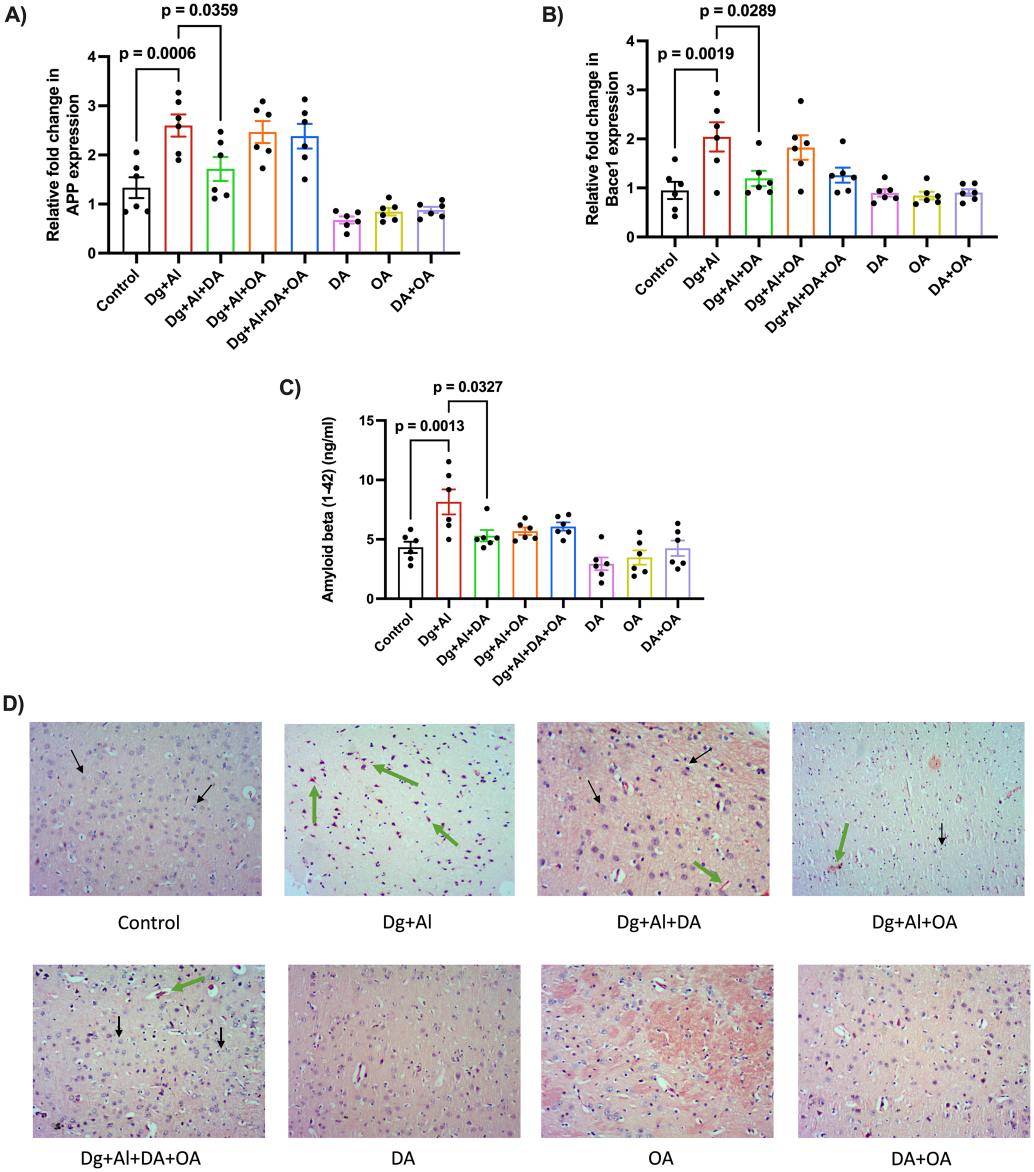
Quantitative and qualitative analysis of amyloid deposition in D-gal combined with AlCl_3_ induced AD-like neurotoxic model: The figure dictates the relative fold change in the mRNA expression of genes responsible for amyloid protein deposition, **(A)** APP and **(B)** BACE1 using RT-PCR. **(C)** Neuronal levels of Amyloid beta (1-42) protein estimated using ELISA. **(D)** Amyloids stained red after using Congo Red staining in cerebral cortex of mice with AD-like neurotoxicity induced by D-gal and AlCl_3_. 10X (Scale-100 μm). Black arrows = nuclei; Green arrows = amyloid deposits. Data is represented as Mean ± SEM (*n* = 6; 3 males, 3 females) and analysed by one-way ANOVA followed by Tukey’s multiple comparison test. Actual *p*-values for all comparisons are displayed. DA, decanoic acid; OA, octanoic acid; Dg, D-galactose, Al, aluminium chloride (AlCl_3_).

The findings reported a significant increase in the mRNA expression of A*β* precursor protein, APP (*p* = 0.0006 versus Control) and amyloidogenic protein, Bace1 (*p* = 0.0019 versus Control), followed by a significant rise in the enzymatic levels of neuronal amyloid-*β* protein (*p* = 0.0013 versus Control) in D-gal/AlCl3 induced AD-like amyloid neurotoxicity (Figures 4A–C). These observations inferred the synthesis of amyloid protein, which was confirmed by the Congo red-stained neuronal cells in the cerebral cortical sections of the neurotoxic mice, as illustrated in Figure 4D. Among the MCT components, only decanoic acid was identified to mitigate the deposition of amyloid protein, as observed by the significant lowering of mRNA expression of APP (*p* = 0.0359 versus Dg + Al) and Bace1 (*p* = 0.0289 versus Dg + Al), followed by the enzymatic levels of A*β* protein (*p* = 0.0327 versus Dg + Al).

### 3.5 Effects of decanoic acid, octanoic acid and their combination on the relative expression of mTOR and AMPK using RT-PCR and ELISA

**Figure 5:**
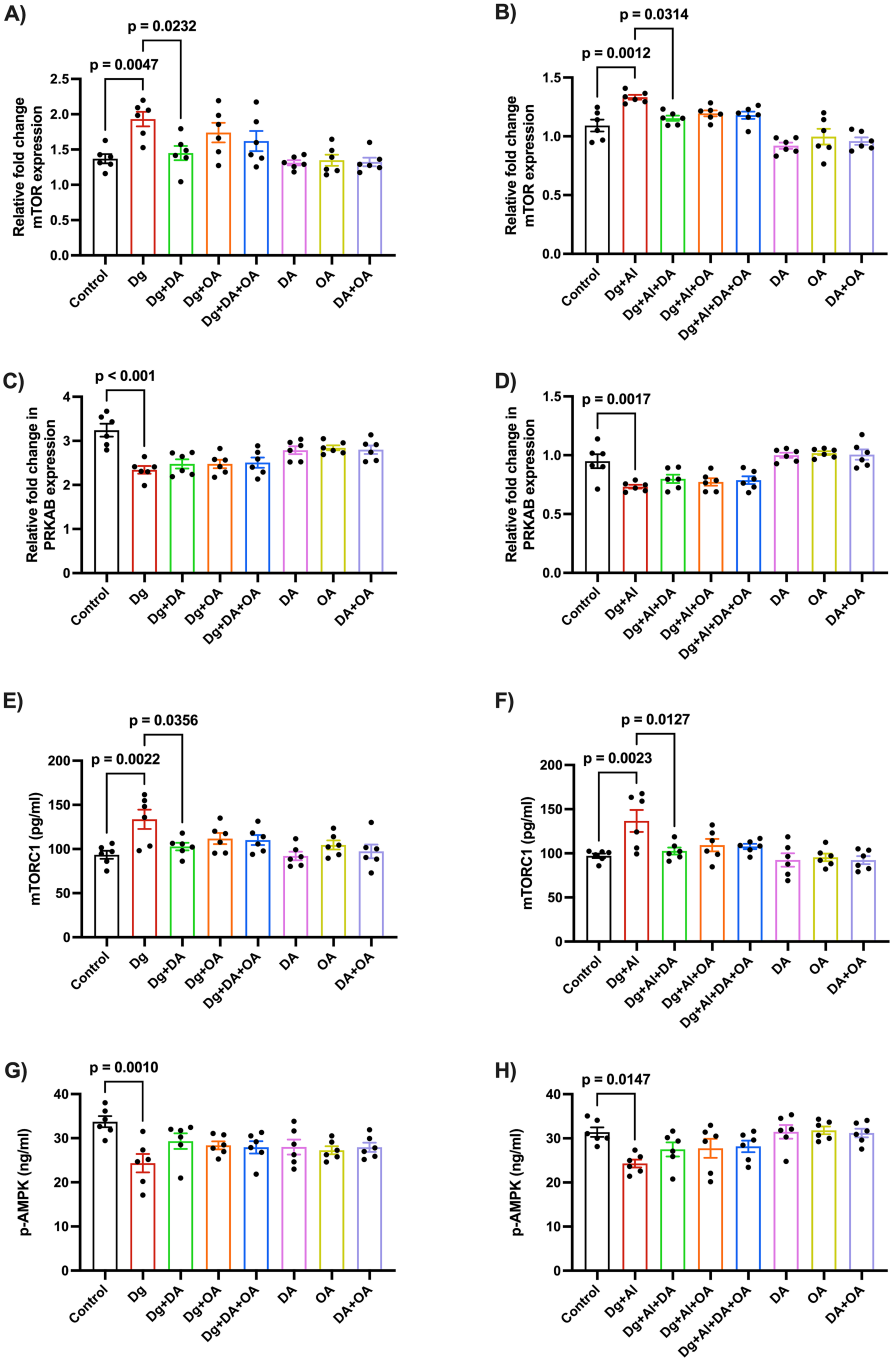
Relative expression of mTOR and p-AMPK using RT-PCR: The figure states the effects of MCT components on the relative fold change in the mRNA expression of **(A,B)** mTOR and **(C,D)** PRKAB expression in the D-gal induced aging model and in the D-gal/AlCl_3_ induced age-related AD-like neurotoxic model, respectively. Enzymatic levels of **(E,F)** mTOR and **(G,H)** AMPK were also determined by ELISA. Data is represented as Mean ± SEM (*n* = 6; 3 males, 3 females) and analysed by one-way ANOVA followed by Tukey’s multiple comparison test. Actual *p*-values for all comparisons are displayed. DA, decanoic acid; OA, octanoic acid; Dg, D-galactose; Al, aluminium chloride (AlCl_3_).

To study the relative fold change in the mRNA expression of mTOR and AMPK through RT-PCR, the primers were derived from the mTOR gene and the PRKAB gene, the beta-regulatory subunit of AMPK, respectively (Table 3). We observed a significant upregulation in both mRNA expression (*p* = 0.0047 versus Control; *p* = 0.0012 versus Control, respectively), and enzymatic activity of mTOR kinase (*p* = 0.0022 versus Control; *p* = 0.0023 versus Control, respectively), accompanied by a significant downregulation in the mRNA expression of AMPK gene marker, PRKAB (*p* < 0.001 versus Control; *p* = 0.0017 versus Control, respectively), and its cerebral enzyme levels (*p* = 0.001 versus Control; *p* = 0.0147 versus Control, respectively), in both accelerated aging model as well as in the AD-like neurotoxic model, respectively (Figure 5). Only decanoic acid was witnessed to significantly inhibit the relative mRNA expression and the enzymatic activity of mTOR kinase in both models of aging (*p* = 0.0232 versus Dg; *p* = 0.0356 versus Dg, respectively) and age-related neurotoxicity (*p* = 0.0314 versus Dg + Al; *p* = 0.0127 versus Dg + Al, respectively).

### 3.6 Effects of decanoic acid, octanoic acid and their combination on senescent *β*-galactosidase activity

**Figure 6:**
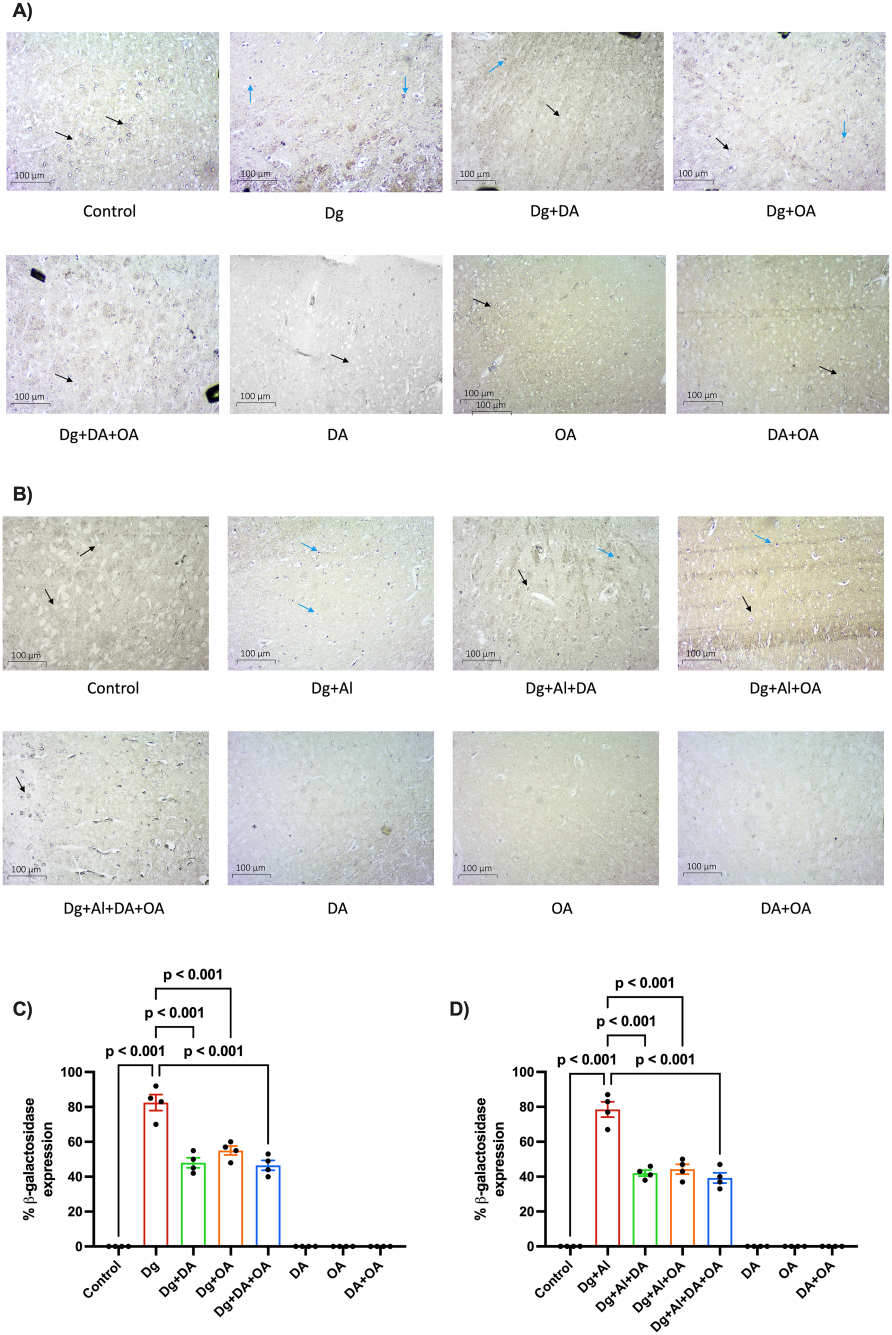
Senescence-associated *β*-galactosidase activity: The figure demonstrates the **(A,B)** microscopic images of the cerebral cortex and **(C,D)** the percentage of senescent positive cells in the D-gal induced aging model and in the D-gal/AlCl_3_ induced age-related AD-like neurotoxic model, respectively. 10X (Scale-100 μm). Black arrows = unstained cells, blue arrows = blue stained (senescent cells). Data is represented as Mean ± SEM (*n* = 2; 4 non-overlapping fields ~100 cells/field) and analysed by one-way ANOVA followed by Tukey’s multiple comparison test. Actual *p*-values for all comparisons are displayed. DA, decanoic acid; OA, octanoic acid; Dg, D-galactose; Al, aluminium chloride (AlCl_3_).

Cellular senescence was detected based on the ability of cells to stain positive for senescence-associated *β*-galactosidase activity, though they cease to replicate. Under an optical microscope, SA-*β*-gal-positive cells (blue-stained) were significantly present in the cortical sections of the brain obtained from mice with induced accelerated aging (*p* ≤ 0.0001 versus Control) and with induced AD-like neurotoxicity (*p* ≤ 0.0001 versus Control). On administration with the MCT dietary components, the percentage of *β*-galactosidase positive cells was significantly suppressed (*p* < 0.001 versus Dg; *p* < 0.001 versus Dg + Al, respectively) as observed in Figure 6.

## 4 Discussion

Medium-chain triglycerides (MCT) supplemented ketogenic diet was introduced as a therapeutic intervention against drug-resistant epilepsy (Schoeler et al., 2021), however, emerging evidence has highlighted its neuroprotective potential against dementia in elderly patients with Alzheimer’s disease and cognitive impairment associated with natural aging (Dunn et al., 2023). MCTs are hydrolysed into medium-chain fatty acids, namely, decanoic acid and octanoic acid, and are readily absorbed in the gastrointestinal tract. These fatty acids are subsequently metabolised through *β*-oxidation in the liver, resulting in ketone metabolites such as acetoacetate, beta-hydroxybutyrate (*β*HB), and acetone, which then circulate systemically (Augustin et al., 2018). Decanoic acid and octanoic acid, along with their ketone body metabolites, cross the blood–brain barrier and can attain neuronal concentrations of up to 50% of plasma levels, providing an additional energy source for neurons and astrocytes (Augustin et al., 2018). Additionally, published literature shows that octanoic (C8) and decanoic (C10) acids have low levels of acute and systemic toxicity. The oral LD₅₀ in rats is higher than 2,000–5,000 mg/kg, and EFSA assessments indicate no genotoxicity or safety concerns related to fatty acids and MCT oils (Mortensen et al., 2017). Long-term rodent studies primarily involving C8/C10 MCTs indicate normal growth patterns and no significant organ toxicity. Additionally, clinical data confirm that these are well tolerated in humans (Traul et al., 2000).

**Figure 7:**
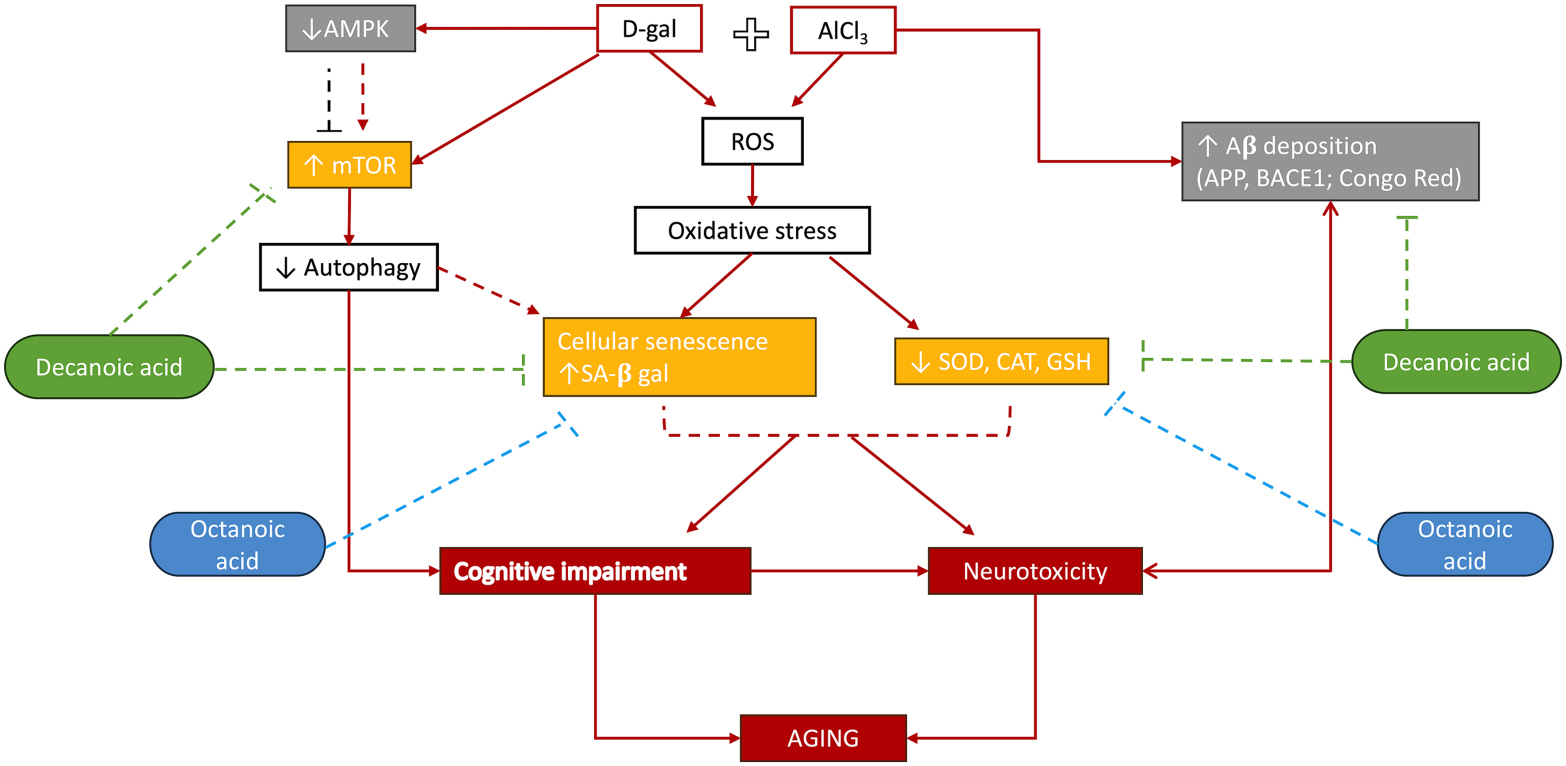
Schematic summary of mechanistic outcomes: D-galactose-induced toxicity increases ROS levels. The antioxidant activity of GSH, SOD, and CAT is reduced. Elevated ROS inhibits AMPK activation and boosts mTOR expression. Deregulated autophagy and oxidative stress promote cellular senescence. Decanoic acid (DA) influences mTOR activity, senescence, and antioxidant defences, while octanoic acid (OA) enhances antioxidant activity and decreases cellular senescence. D-galactose combined with aluminium chloride (AlCl3) causes AD-like amyloid toxicity, which is reduced by decanoic acid but not by octanoic acid.

Decanoic acid and octanoic acid are medium-chain fatty acids that constitute the MCT diet; however, no study has been performed till now to evaluate their individual effects against age-related pathological markers or neurotoxicity. In this work, we report for the first time the distinct neuroprotective potential of decanoic acid against cognitive impairment associated with D-gal induced accelerated aging model and age-related neurotoxicity induced by D-gal combined with AlCl_3_. These effects were shown to be mediated by altering aging hallmarks such as stimulating the antioxidant activity of sodium dismutase and catalase enzyme; promoting autophagy by inhibiting mTOR expression; and repressing cellular senescence by decreasing the incidence of *β*-galactose-positive stained cells (Figure 7). Octanoic acid, however, when administered individually, only demonstrated positive effects against neurobehavioural cognitive impairment, possibly by increasing the antioxidant activity of sodium dismutase enzyme, and reducing the number of *β*-galactose-stained senescent cells. Only when combined with decanoic acid, octanoic acid exhibited positive results in altering the biomarkers evaluating the hypothesised mechanisms. The present study, thereby, proposes differential profiles and mechanisms for the two MCT diet components.

Several clinical investigations have shown positive effects of MCT diet on cognitive functions in patients with mild cognitive impairment and mild to moderate Alzheimer’s disease (Fortier et al., 2019; Xu Q. et al., 2020). For instance, a clinical study reported that chronic administration of an MCT diet formula resulted in constructive effects on verbal memory and processing speed in patients with Alzheimer’s disease (Ota et al., 2019). We observed alleviation of regressed spatial memory and learning associated with accelerated aging as well as age-related neurotoxic model on administration with major MCT dietary components, decanoic acid, octanoic acid and their combination in mice. These results support the previous findings observed in naturally aged rats, where administration of decanoic acid and octanoic acid led to improved social recognition and synaptic elasticity (Wang and Mitchell, 2016). An alternative preclinical evaluation in rats, which studied the effects of ketosis induced by MCT supplementation, also showed enhanced working memory (Shcherbakova et al., 2022).

The deterioration of cognitive function associated with accelerated aging induced by chronic delivery of exogenous D-galactose (D-gal) was accompanied by an increase in oxidative burden (Shwe et al., 2018). Elevated oxidative stress is also linked with a lowered cognitive function and can be considered an early biomarker for memory loss in the elderly (Hajjar et al., 2018; Kandlur et al., 2020). Hence, our study reported a remarkable decrease in the activity of the anti-oxidant enzymes- glutathione (GSH), catalase (CAT) and superoxide dismutase (SOD) in D-gal and D-gal/AlCl_3_ induced neurotoxic models. Our results are supported by previous *in vivo* studies that illustrate the effects of D-gal on pro-oxidant/anti-oxidant balance (Haider et al., 2015) and decline in the anti-oxidant activity induced by chronic co-administration of D-gal/AlCl_3_ (Hu et al., 2022). On the contrary, the MCT ketogenic diet has been known to reverse oxidative stress and the synthesis of ROS (Zhang et al., 2023; Schuck et al., 2009). The results from our study further endorse the anti-oxidant properties of the MCT dietary components in both the accelerated aging model and the age-related neurotoxic model. Decanoic acid, octanoic acid and their combination exhibited reversal of the decline in the SOD enzymatic action; however, only decanoic acid increased the loss in catalase activity as observed in both the neurotoxic models. This outcome can be explained by a previous study where decanoic acid reduced the release of hydrogen peroxide (H_2_O_2_) in neuronal cells, thereby increasing the decomposition by the catalase enzyme (Mett and Müller, 2021). The excessive mitochondrial H_2_O_2_ is also associated with accelerating aging (Giorgio et al., 2007) and A*β* toxicity in Alzheimer’s disease (Milton, 2004). Hence, the underlying mechanism of the observed neuroprotective effects of MCT dietary components can be linked to their antioxidant properties.

Amyloid toxicity is identified by the accumulation of amyloid proteins in the form of plaques. Neuronal aggregation of amyloid-*β* peptide is the cause of dementia associated with Alzheimer’s disease (Ma et al., 2022). As observed in our study, increased mRNA expression of amyloidogenic protein, BACE1 and A*β* precursor protein, APP, can be correlated to the increase in the synthesis of neuronal amyloid-*β* protein (Jain et al., 2024). The established outcomes were supported by available pre-clinical studies that report the incidence of amyloid-*β* protein in the D-gal/AlCl_3_ induced model (Luo et al., 2009; Wei et al., 2017; Sun et al., 2009). Among the MCT components, decanoic acid was identified to mitigate the levels of amyloid protein as observed by the lowering of mRNA expression of APP, followed by the enzymatic levels of A*β* protein. Our results are supported by the emerging clinical evidence that has recounted positive effects of MCT dietary supplements against dementia associated with Alzheimer’s disease (Ota et al., 2019; Juby et al., 2022; Castro et al., 2023). Furthermore, it was also discovered that coconut oil, from which decanoic acid and octanoic acid are derived, attenuated the effects of amyloid-*β* in cortical neurons by altering mitochondrial functioning and the induction of ketosis (Nafar and Mearow, 2014; Studzinski et al., 2008).

Emerging evidence suggests that there is a decline in autophagic activity with accelerated aging (Aman et al., 2021) and age-related neurodegenerative disorders (Lou et al., 2020). Abundant preclinical experiments performed on *Caenorhabditis elegans*, *Drosophila*, rodents and mammalian cells have proclaimed an impairment in autophagic flux and contribute to the progression of age-related disorders such as Alzheimer’s disease (Simonsen et al., 2008; Sun et al., 2020; Yu et al., 2017; Ott et al., 2016; Lipinski et al., 2010). The core pathways of autophagy, initiated in response to a stress stimulus, are governed by canonical inducers, mechanistic target of rapamycin (mTOR) or 5′ AMP-activated protein kinase (AMPK) (Kim et al., 2011). Our study reported the amplification of mTOR, and reduction of phosphorylated (p-)AMPK enzyme in both accelerated aging model as well as in the Alzheimer’s-like neurotoxic model. Previous *in vivo* experiments have reported similar outcomes about decrease in the induction of autophagy regulated by mTOR and p-AMPK in D-gal (Yang et al., 2022; Ameen et al., 2022) as well as in D-gal/AlCl_3_ induced neurotoxic rodent models (Hu et al., 2024; Ryu et al., 2020). A recent study stated that a diet enriched with high MCT content improves autophagic flux in mice hepatocytes (Wang et al., 2017). In the present investigation, only decanoic acid was witnessed to inhibit the rise in mTOR expression in both models of aging and age-related neurotoxicity. The observed effect is validated by existing literature that supports inhibition of mTOR activity by decanoic acid (Warren et al., 2020). Another preclinical study that employed the *Dictyostelium* model concluded that decanoic acid, instead of octanoic acid, induces autophagy by promoting the expression of autophagy-inducing proteins (Warren et al., 2021). Although, octanoic acid alone failed to demonstrate any positive effects on the core autophagy regulating markers in the present study, evidence suggests that octanoic acid can upregulate autophagy via a different mechanism of inhibiting the c-Jun N-terminal kinase (JNK) pathway in rats (He et al., 2022).

Cellular senescence is achieved when proliferating cells become resistant to growth-promoting factors and exist in a state of stable cell cycle arrest. Increase in senescent cells contributes to the pathophysiological progression of natural aging. Various stressors including mitochondrial insufficiency, increase in reactive oxygen species (ROS), oncogene activation, telomere dysfunction and DNA damage can trigger the state of senescence in a cell (Di Micco et al., 2021). The prevention of neuronal cellular senescence in age-related disorders including Alzheimer’s disease, can also be correlated with the induction of autophagy by inhibiting the mTOR pathway (Aman et al., 2021; Sikora et al., 2021). Activation of senescence-associated *β*-galactosidase (SA-*β*-gal) was introduced as the primary biomarker to detect senescent cells (Dimri et al., 1995). We analysed the incidence of senescent cells through the SA-*β*-gal staining assay. SA-*β*-gal positive cells were remarkably depicted in cortical sections of brain obtained from mice with accelerated aging as well as with induced Alzheimer’s disease neurotoxicity. These observations are in accordance with the previous *in vitro* and *in vivo* investigations that narrated induction of cellular senescence by D-gal (Lu et al., 2022; Zhu et al., 2014; Sun et al., 2018; Xu et al., 2018). AlCl_3_ has also exhibited SA-*β*-gal positive senescent cells in rats (Sentyabreva et al., 2023), whereas study conducted on Zebra Fish model showed senescence due to combined effects of D-gal and AlCl_3_ (Luo et al., 2024). On administration of either or both of the MCT dietary components, the occurrence of senescent cells was restricted during our experimentation. This behaviour has not been reported before and could be explained by their effectiveness in promoting autophagy, thereby reducing cellular senescence. However, evidence exists whereby ketosis or mimicking calorie restriction has also prevented senescence in aging (Aminzadeh-Gohari et al., 2024).

Lastly, we ascertained the production of ketone bodies to determine a correlation between ketosis and the observed neuroprotective effects of decanoic acid and octanoic acid. This was based on preclinical evidence showing enhanced working memory due to ketosis induced by MCT supplementation (Shcherbakova et al., 2022). Interestingly, octanoic acid revealed the highest levels of urinary ketone bodies. However, it failed to express any promising results against the evaluated mechanistic biomarkers. Although, both decanoic acid and octanoic acid possess neuroprotection against cognitive impairment, there is an absolute distinction between their underlying mechanisms and can be described by previous findings that proclaim elevated secretion of the ketone body, *β*-hydroxybutyrate (*β*HB) by octanoic acid (Sonnay et al., 2019) and the fact that octanoic acid undergoes higher *β*-oxidation than decanoic acid in neuronal cells (Khabbush et al., 2017). This notion compels us to believe that octanoic acid might not be directly or independently imposing beneficial pro-cognitive effects but possibly through ketosis.

Another hypothesised mechanism by which decanoic acid might express its positive outcomes through activation of peroxisome proliferator-activated receptor-gamma (PPAR-*γ*) (Malapaka et al., 2012). Modulation of PPAR-γ by an agonist has resulted in delayed aging in mice and improvement in cognitive decline that can be associated with Alzheimer’s disease (Xu L. et al., 2020). Correspondingly, a recent study also identified decanoic acid, but not octanoic acid, as a non-competitive antagonist of AMPA-type glutamate receptors responsible for inhibition of excitatory synapses in an *in vitro* seizure model (Chang et al., 2015). AMPA receptors are responsible for mediating excitatory synaptic transmission and hence, their overexcitation causes excitotoxicity implied in neurodegenerative disorders including Alzheimer’s disease (Chang et al., 2015).

Despite exposing the clinically relevant promising neuroprotective effects of MCT dietary components, our study had several limitations. On inspection of the available literature, contradicting negative data were discovered associated with the MCT component. Research involving mice has shown a notable decline in bone health following prolonged use of MCT components, particularly octanoic acid (Jain and Vohora, 2025; Jain et al., 2021). Our study did not measure the amount of food intake or its impact. While the present study investigated the mRNA expression of key signalling molecules, future research is required to confirm these findings at the protein level using methods such as Western blot or other proteomic techniques to strengthen mechanistic insights. This study is the first to identify the senescence-related effects of decanoic acid and octanoic acid by measuring SA-*β*-galactosidase activity, a primary indicator of senescent cells. Future studies should include additional markers such as p21, p16, and Ki67 to reinforce and validate these preliminary findings. Moreover, they should also measure the A*β* 1–42 to A*β* 1–40 ratio for a more comprehensive understanding of amyloid deposition. Although D-gal-induced aging models effectively simulate accelerated aging and neurotoxicity similar to age-related Alzheimer’s disease, future research should consider using naturally aged mice or transgenic models like SAMP8, APP/PS1, and 5xFAD mice. Future research could focus on biochemical analyses of specific regions, particularly the hippocampus.

Nevertheless, ours is the only study that clarified the distinctive role of individual components of MCT diet on neurotoxicity associated with aging and age-related neurodegeneration, providing a better understanding of their respective underlying mechanisms for future perspective studies.

## 5 Conclusion

In conclusion, the findings of the present study highlights the neuroprotective potential of decanoic acid, on cognitive impairment, and neurotoxicity associated with D-gal and D-gal/AlCl3 treated mice possibly through modulation of aging hallmarks, specifically, by enhancing antioxidant activity (GSH, SOD, CAT), promoting autophagy by regulating nutrient sensing pathways, primarily inhibiting mTOR expression and improving enzymatic levels of p-AMPK, and reducing the frequency of senescence-associated *β*-galactosidase positive neuronal cells. Decanoic acid also inhibited the serum enzymatic activity of amyloid-*β*, and reduced amyloid deposits in the brain as observed through Congo Red staining. On the contrary, although octanoic acid exhibited positive effects alone against memory deficit, cellular senescence, and amyloid deposition, its effectiveness in regulating the hypothesised mechanisms was observed only when combined with decanoic acid. The combined MCT components also expressed anti-amyloidogenic potential against the D-gal/AlCl_3_-induced neurotoxic model. This indicate a differential profile of the two MCT diet components suggesting researchers to further modify the MCT diet composition as per its therapeutic indication as it is being actively investigated as preventive tool against aging, and other metabolic and neurodegenerative disorders including Alzheimer’s disease, drug-resistant epilepsy, Parkinson’s disease and amyotrophic lateral sclerosis (ALS), deliberating their advantages and disadvantages. Therefore, this field of research integrating the effects of individual components of the MCT diet, namely decanoic acid and octanoic acid, remains uncharted and needs further rigorous investigation.

## Data Availability

The raw data supporting the conclusions of this article will be made available by the authors, without undue reservation.

## Footnotes

^1^ http://www.ncbi.nlm.nih.gov/tools/primer-blast/

## Glossary

AlCl_3_: Aluminium chloride
AMPK: Adenosine monophosphate-activated protein kinase
ANOVA: analysis of variance
APP: Amyloid precursor protein
A*β*: Amyloid beta
BACE1: Beta-secretase 1
Bax: Bcl-2-associated protein x
Bcl2: B-cell leukaemia/lymphoma 2
CAT: Catalase
Dg/D-gal: D-galactose
DA: Decanoic acid
ELISA: Enzyme-linked immunosorbent assay
GSH: Reduced Glutathione
MCT: Medium-chained triglycerides
mTOR: Mammalian target of rapamycin
OA: Octanoic acid
RT-PCR: Reverse transcription polymerase chain reaction
SA-*β* gal: Senescence *β*-Galactosidase
SEM: Standard error of the mean
SOD: Superoxide dismutase
Trp53: Transformation-related protein 53
