# Supplementary material for: Decanoic acid, an MCT dietary component, alleviates cognitive impairment, cellular senescence, and promotes autophagy in accelerated aging and neurotoxic mouse models induced by chronic administration of D-galactose and D-galactose/AlCl3
Source: Front Aging Neurosci. 2025 Nov 19;17:1676926. doi: 10.3389/fnagi.2025.1676926 (PMC12672871; doi:10.3389/fnagi.2025.1676926)
Supplement: Supplementary file 1 [file Data_Sheet_1.pdf]

Decanoic acid, an MCT dietary component, alleviates cognitive impairment, cellular senescence, and promotes autophagy in accelerated aging and neurotoxic mouse models induced by chronic administration of D-galactose and D-galactose/AlCl3.

Shreshta Jain[1], Divya Vohora[1]

SUPPLEMENTARY MATERIAL

Table S1. Numerical data (Mean ± SEM) of the evaluated parameters of D-gal-induced and D-gal/$AlCl_3$-induced models-

D-gal-induced accelerated aging model								
	**Control**	**Dg**	**Dg+DA**	**Dg+OA**	**Dg+DA+ OA**	**DA**	**OA**	**DA+ OA**
Oxidative stress								
SOD	195.1± 18.83	86.66± 10.76***	185.0± 17.21##	160.3± 16.19#	161.2± 15.50#	181.2± 16.84	170.0± 13.52	160.6± 18.91
CAT	3.045± 0.1969	1.773± 0.1658***	2.606± 0.1929#	2.176± 0.1863	2.085± 0.1717	2.634± 0.1581	2.730± 0.1464	2.590± 0.2359
GSH	246.3± 17.24	152.7± 12.08**	191.7± 6.565	164.2± 12.93	198.4± 6.098	268.1± 12.90	244.2± 17.56	251.8± 25.02
Autophagy								
mTOR mRNA	1.370± 0.06425	1.932± 0.1032 **	1.450± 0.1009#	1.740± 0.1385	1.621± 0.1428	1.312± 0.0367	1.348± 0.0796	1.324± 0.0608
mTOR enzyme	93.45± 4.602	133.7± 10.99 **	102.8± 4.277#	111.8± 6.310	110.2± 5.677	92.08± 4.816	104.6± 5.150	97.33± 7.796
PRKAB mRNA	3.243± 0.1459	2.343± 0.08758***	2.477± 0.1032	2.476± 0.09495	2.507± 0.1155	2.789± 0.0902	2.842± 0.0563	2.800± 0.0963
AMPK enzyme	33.74± 1.259	24.34± 2.090 ***	29.33± 1.770	28.38± 0.9093	27.93± 1.389	28.00± 1.702	27.27± 0.9014	27.95± 1.039

D-gal/AlCl3-induced Alzheimer's disease-like neurotoxic model								
	**Control**	**Dg+Al**	**Dg+Al+ DA**	**Dg+Al+ OA**	**Dg+Al+ DA+OA**	**DA**	**OA**	**DA+ OA**
Oxidative stress								
SOD	116.4± 11.16	63.73± 11.43***	96.69± 3.928^	86.48± 3.271	97.23± 5.121^	109.8± 4.017	99.94± 9.386	96.09± 2.762
CAT	2.674± 0.1557	1.733± 0.1093**	2.465± 0.2395^	2.031± 0.1646	2.280± 0.1748	2.480± 0.0879	2.392± 0.0696	2.604± 0.1386
GSH	267.4± 24.87	153.1± 13.40**	218.5± 18.89	209.4± 17.30	212.8± 11.63	252.9± 17.32	244.2± 17.46	253.5± 17.96
Autophagy								
mTOR mRNA	1.092± 0.05016	1.333± 0.01976**	1.154± 0.02190^	1.195± 0.02562	1.180± 0.03132	0.920± 0.0264	0.997± 0.0668	0.959± 0.0313
mTOR enzyme	97.15± 2.581	136.6± 12.47**	102.6± 3.974^	109.3± 7.043	107.8± 3.015	92.43± 7.598	95.42± 4.290	92.29± 4.510
PRKAB mRNA	0.949± 0.05890	0.7327± 0.01606**	0.7992± 0.03497	0.7722± 0.03148	0.7883± 0.03290	0.997± 0.0215	1.018± 0.0156	1.005± 0.0432
AMPK enzyme	31.40± 11.084	24.30± 0.8906*	27.49± 1.590	27.74± 2.159	28.16± 1.316	31.47± 1.538	31.81± 0.8616	31.21± 0.9853
Amyloid toxicity								
APP	1.334± 0.2122	2.600± 0.2257***	1.716± 0.2426^	2.466± 0.2230	2.382± 0.2494	0.675± 0.0726	0.846± 0.0762	0.880± 0.063
Bace1	0.951± 0.1748	2.043± 0.2984**	1.195± 0.1572^	1.825± 0.2492	1.261± 0.1541	0.900± 0.0753	0.845± 0.0783	0.906± 0.0674
$A\beta_{1-42}$	4.332± 0.4737	8.151± 1.057**	5.306± 0.4746^	5.681± 0.3119	6.079± 0.3449	2.935± 0.5376	3.471± 0.6012	4.251± 0.6504

Data is represented as Mean ± SEM and analysed by one-way ANOVA followed by Tukey's multiple comparison test. The significance is ascertained as *p < 0.05, **p<0.01 ***p<0.001 versus Control; #p<0.05, ##p<0.01, ###p<0.001 versus Dg; ^p<0.05, ^^p<0.01, ^^^p<0.001 versus Dg+Al

DA- Decanoic acid; OA- Octanoic acid; Dg- D-galactose; Al- Aluminium chloride ($AlCl_3$)
